# AGEMAP: A Gene Expression Database for Aging in Mice

**DOI:** 10.1371/journal.pgen.0030201

**Published:** 2007-11-30

**Authors:** Jacob M Zahn, Suresh Poosala, Art B Owen, Donald K Ingram, Ana Lustig, Arnell Carter, Ashani T Weeraratna, Dennis D Taub, Myriam Gorospe, Krystyna Mazan-Mamczarz, Edward G Lakatta, Kenneth R Boheler, Xiangru Xu, Mark P Mattson, Geppino Falco, Minoru S. H Ko, David Schlessinger, Jeffrey Firman, Sarah K Kummerfeld, William H Wood, Alan B Zonderman, Stuart K Kim, Kevin G Becker

**Affiliations:** 1 Department of Developmental Biology, Stanford University Medical Center, Stanford, California, United States of America; 2 National Institute on Aging, National Institutes of Health, Baltimore, Maryland, United States of America; 3 Department of Statistics, Stanford University, Stanford, California, United States of America; 4 Department of Genetics, Stanford University Medical Center, Stanford, California, United States of America; The Jackson Laboratory, United States of America

## Abstract

We present the AGEMAP (Atlas of Gene Expression in Mouse Aging Project) gene expression database, which is a resource that catalogs changes in gene expression as a function of age in mice. The AGEMAP database includes expression changes for 8,932 genes in 16 tissues as a function of age. We found great heterogeneity in the amount of transcriptional changes with age in different tissues. Some tissues displayed large transcriptional differences in old mice, suggesting that these tissues may contribute strongly to organismal decline. Other tissues showed few or no changes in expression with age, indicating strong levels of homeostasis throughout life. Based on the pattern of age-related transcriptional changes, we found that tissues could be classified into one of three aging processes: (1) a pattern common to neural tissues, (2) a pattern for vascular tissues, and (3) a pattern for steroid-responsive tissues. We observed that different tissues age in a coordinated fashion in individual mice, such that certain mice exhibit rapid aging, whereas others exhibit slow aging for multiple tissues. Finally, we compared the transcriptional profiles for aging in mice to those from humans, flies, and worms. We found that genes involved in the electron transport chain show common age regulation in all four species, indicating that these genes may be exceptionally good markers of aging. However, we saw no overall correlation of age regulation between mice and humans, suggesting that aging processes in mice and humans may be fundamentally different.

## Author Summary

This work studies the aging process in mice using DNA microarrays to identify genes that change expression during aging. The entire set of age-regulated genes constitutes a transcriptional profile that can be used to measure ages of different individuals. Furthermore, the aging expression profile highlights genetic and metabolic pathways that change with age, providing key insights about possible molecular changes that may contribute to cell senescence and physiological decline. This aging study is massive in scope, involving gene expression measurements from 16 different tissues at four different ages. Expression data for genes of interest can be queried over the web by researchers interested in aging. Different tissues were found to have strikingly different levels of age-related change, and could be divided into three groups based on their patterns of aging: a neural group, a vascular group, and a steroid-responsive group. The electron transport chain pathway stands out because it is the only genetic pathway that shows a similar pattern of age-related change in mice, humans, worms, and flies. However, there is little overall similarity between changes in gene expression during aging of humans and mice, consistent with evolutionary theories suggesting that aging lies outside the force of natural selection.

## Introduction

Aging is characterized by the progressive functional decline of multiple organs and tissues, eventually culminating in death. A long sought after goal has been to identify biomarkers of aging to characterize aging. Biomarkers of aging may indeed be tightly linked to aging processes, and hence may provide insight regarding mechanisms underlying aging. Changes in cellular morphology or cell type number are quantifiable traits that can be correlated with physiological age. For example, age-related changes in specific tissues include hair whitening, muscle atrophy, wrinkling of skin, thymic involution, and glomerulosclerosis of the kidney. However, these age-related tissue changes do not pinpoint specific genes or molecular pathways involved in distinct aging processes. Further, it is difficult to determine whether age-related changes in different tissues are due to common or distinct molecular causes. At the molecular level, an attractive approach is to use changes in gene expression as biomarkers of aging. DNA arrays can be used to scan a large fraction of the genome for genes that change expression with age. Identification of age-regulated genes provides key insights regarding aging.

In mice, at least 19 studies have profiled changes in gene expression with age in nine separate mouse tissues. Many of these studies find that similar classes of genes are regulated with age in different tissues. For instance, genes involved in the inflammatory response and heat shock factors have been found to increase expression levels with age in mouse brain, muscle, and heart [1–5]. Other pathways commonly found to be associated with aging in mice include metabolic energy pathways [1,6], extracellular matrix genes [5,7], and degradation pathways [1,4,5,8]. These studies also show that feeding mice under conditions of caloric restriction attenuates many age-related changes in gene expression.

These DNA microarray studies were performed using different experimental protocols, with different types of gene arrays (e.g., Affymetrix GeneChips or DNA microarrays), and by different labs. Because of these differences in methodology, most studies in mouse aging have not compared their results to each other. In this study, we present AGEMAP (Atlas of Gene Expression in Mouse Aging Project), which is a highly standardized study of gene expression changes as a function of age in mice. AGEMAP includes expression data for 8,932 genes from 16 tissues taken from the same set of C57BL/6 mice using an identical gene array platform and experimental protocol. Because the gene array experiments were analyzed at the same time and in the same way, we can compare gene expression profiles for aging between different tissues in order to identify common aging biomarkers.

Previous studies have shown that the overall pattern of expression from age-regulated genes (a gene expression profile) can be used as a biomarker of aging. For instance, a transcriptional profile of aging in human kidneys correlated strongly with a histological measure of kidney function, and thus could be used to predict physiological age [9]. Similarly, in human muscle, a gene expression profile of aging was found to correlate with Type II muscle fiber atrophy, a measure of muscular age [10]. In mice, changes in gene expression levels associated with aging were seen to be slowed by caloric restriction, a known aging intervention [1,2].

Expression profiles can be used to compare aging in different tissues. In humans, there is extensive overlap in gene expression profiles for aging between different parts of the same tissue. Specifically, similar aging transcriptional profiles were found between the cortex and medulla of the kidney and between different sections of the prefrontal cortex of the brain [9,11]. In contrast, there is little in common between aging expression profiles across different human tissues. However, a small amount of overlap was found involving six genetic pathways that showed similar age regulation among the kidney, skeletal muscle, and brain [10]. These six common age-regulated genetic pathways may be closely tied to basic processes of human aging. Lessons learned about the role of these pathways in aging may reveal not only how tissues grow old, but also how aging results in pathology, disease, and loss of function in elderly individuals.

Expression profiles of aging for one species can be compared to that of another species, thereby showing which age-related changes in expression are unique to one species and which are shared by different animals [12]. Genes and gene pathways that appear to be age regulated in diverse species may be unavoidably linked with major aging processes, making them exceptionally strong candidates for biomarkers of aging. Comparisons of transcript profiles of aging among human, mouse, fly, and worm have shown an overall decrease in the expression level of the electron transport chain genes in these distantly related species [10,13]. The mitochondrial electron transport chain is the primary source for free radical production, which can result in cellular damage. Alteration in the expression of the electron transport chain with age likely affects levels of oxidative damage.

In this work, we present an analysis of data from AGEMAP, a database of changes in gene expression with respect to age in mice, which can be used as a resource for gerontological research. We generated mRNA transcript profiles of aging involving 8,932 genes and 16 mouse tissues at four times during aging. The raw data can be downloaded and reanalyzed by researchers interested in specific tissues or genes. We show that the entire gene expression profile of aging can be analyzed as a molecular phenotype of aging. First, we used the number of age-regulated genes in different tissues to estimate the magnitude of age-related decline among tissues and found that there were great differences in the levels of age-related change in different tissues. Some tissues showed little or no changes in expression (e.g., liver and striatum), whereas other tissues showed a great deal of transcriptional change with aging (e.g., thymus). Second, the aging expression profile for different tissues showed three main patterns for murine aging (neural, vascular, and steroid-responsive). Finally, we compared the expression profiles for mouse aging to those from human aging. Although there are a small number of genetic pathways that age similarly between the two organisms, we found no overall correlation in age-related transcriptional changes between mouse and human.

## Results

### Gene Expression Profiles of Aging in 16 Mouse Tissues

We profiled the effects of aging on gene expression in different tissues dissected from C57BL/6 mice. Mice were of ages 1, 6, 16, and 24 mo, with ten mice per age cohort (five mice of each sex). We dissected 16 tissues from each mouse: the cerebellum, cerebrum, striatum, hippocampus, spinal cord, adrenal glands, heart, lung, liver, kidney, muscle, spleen, thymus, bone marrow, eye, and gonads. These particular tissues were surgically accessible and relatively easy to harvest in the limited amount of time available before the onset of tissue ischemia. We isolated mRNA from each tissue sample and generated radiolabeled cDNA, which was then hybridized to two filter membranes containing a total of 16,896 cDNA clones corresponding to 8,932 genes. Data from 23 arrays were eliminated for quality-control reasons. In total, we generated expression data from 617 gene arrays including 16 tissues and four ages. The full set of expression data from these experiments can be found in Table S1, and investigators can display age-related expression data for specific genes of interest at http://cmgm.stanford.edu/~kimlab/aging_mouse.

First, we wanted to identify genes that showed age-related changes in each tissue. We employed a multiple regression model taking age and sex into account in order to calculate the slope of expression with age for each of the 8,932 genes on the gene array (Materials and Methods). The 16 tissues showed from 0 to 346 age-regulated genes (*p* < 0.001) (Table S2). We used two criteria to determine whether the amount of age-regulation observed for each tissue was statistically significant. First, we restricted attention to tissues with more than nine significantly age-regulated genes, because we would expect to see nine genes by chance alone at *p* < 0.001. Second, we made sure that the actual number of identified genes was unusually large compared to what we would see if there were no age-regulated genes, while accounting for the possibility of correlations existing between the expression levels of different genes. To do this, we permuted the ages of the male mice and those of the female mice and repeated the tests. In 1,000 independent random permutations of this kind, we counted how often we obtained more significant genes in the permuted dataset than in the original one. We discarded data from tissues if more than 10% of permutations gave rise to a greater number of apparently age related genes than we found for a given tissue. Of the 16 tissues, six were eliminated by the first test, one more by the second, and nine showed statistically significant age regulation (Tables 1 and S2).

**Table 1:**
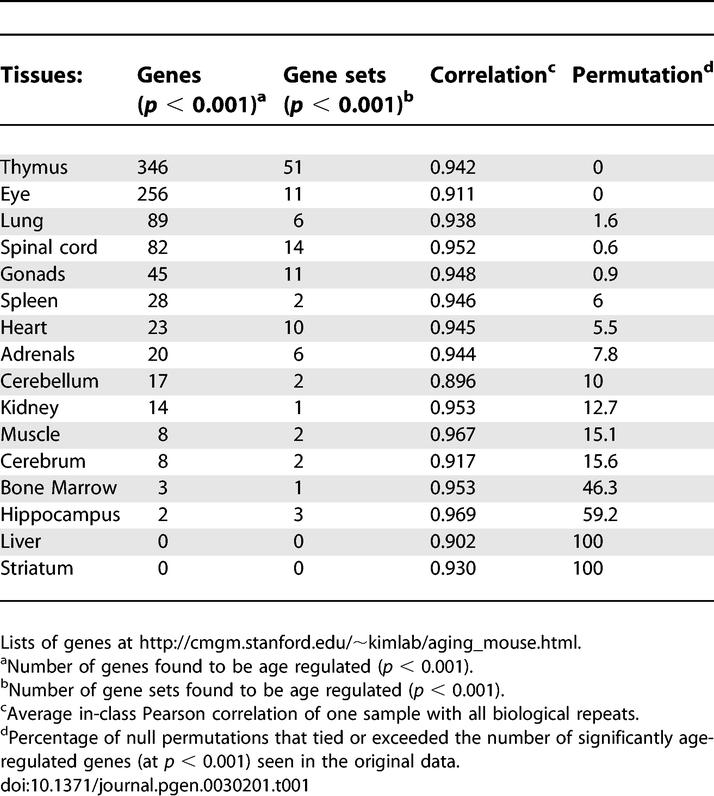
Age-Regulated Genes

One of the tissues (gonads) consists of 20 ovary samples and 20 testes samples. When the ovary and testes samples were analyzed separately, we did not observe a significant number of age-regulated genes, possibly because the sample size is smaller than the other tissues. However, surprisingly, when we grouped the ovaries and testes together, we found a relatively high number of age-regulated genes (45 genes, *p* < 0.001), indicating that these germline tissues share a common underlying aging profile. Age-related expression changes are relatively small in magnitude; most genes increase or decrease expression less than 2-fold over the course of life. The remaining seven tissues showed little or no age-dependent expression changes.

We also explored the impact of aging on the overall expression levels of gene sets and molecular pathways within mouse tissues. We used a modified gene set enrichment analysis [14,15], which measures the cumulative effect of small but consistent changes in expression levels of genes within a gene set. This method is very sensitive, as it can show whether overall age regulation of a gene set is statistically significant even when expression changes of individual genes that comprise the gene set are not. We assayed a collection of 401 gene sets consisting of between ten and 200 genes defined by the Gene Ontology Consortium for age regulation (Table S3) [16]. We used gene set enrichment analysis to assay the age regulation of these 401 gene sets in each of the 16 mouse tissues. Our version of gene set enrichment analysis employs a Van der Waerden statistic to determine whether a gene set is skewed towards an overall increasing or decreasing expression level with age, compared to a random, identically sized set of genes (*p* < 0.001). Our analysis makes 401 hypotheses, one for each gene set. For each hypothesis, we get a van der Waerden test statistic and judge its significance in a bootstrap analysis. This analysis models the mice as randomly sampled. The gene sets themselves are treated as fixed, and the bootstrap analysis assigns *p-*values based on resampling of mice without assuming that the gene set was a random sample. Our van der Waerden statistic is a special case of the test statistic advocated by Newton et al. [17]. The thymus showed the greatest number of age-regulated gene sets (51 age-regulated gene sets), whereas the liver and striatum showed none. Of 16 mouse tissues, ten exhibit more age-regulated gene sets than would be expected by chance alone; nine of these tissues also have a statistically significant number of age-regulated genes (Tables 1 and S4).

Only nine of the 16 tissues showed significant levels of age-related gene expression changes. To test whether different levels of age regulation reflect different quality of expression data from the gene filters, we calculated the average Pearson correlation for all genes between biological repeats. We found no association between experimental variability and the number of age-regulated genes found in different tissues (Table 1), suggesting that the difference in number of age-regulated genes in each tissue is due to differences in the tissues themselves rather than differences in technical reproducibility. Thus, tissues may not all age at the same rate. Some tissues may age rapidly, while others remain relatively constant throughout life. For instance, the histological appearance of the liver remains relatively constant throughout life and likewise we found no discernable changes in gene expression when assayed by cDNA arrays. Conversely, sections of the thymus show obvious histological changes (thymic involution) over the course of life [18], and there are a large number of age-regulated genes in this tissue. Thus, the number of age-regulated genes in a tissue may be a measure of the amount of change in that tissue during aging.

### Common Patterns of Aging in Multiple Tissues

We were interested in finding which genes and gene sets are similarly age-regulated in multiple mouse tissues, which could potentially identify core cell biological pathways involved in common aging mechanisms. We used empirical meta-analysis to find genes, as well as gene sets that are age regulated across multiple tissues. Empirical meta-analysis combines single *p*-values for age regulation of a gene or gene set from each tissue into an overall *p*-value representing age regulation in all of the tissues (Materials and Methods). We identified 314 genes that were significantly age regulated in multiple tissues (*p* < 0.001) and in which no single tissue contributed more than 50% of the test statistic's value (Table S5). Given 8,932 genes, we would expect nine genes or less to appear age regulated in multiple tissues at this threshold.

We also investigated the false discovery distribution by permuting ages for all samples 1,000 times and recalculating empirical meta-analysis *p*-values, maintaining permuted ages for the same mouse within every tissue. We found that the average number of genes returned by permutation in this simulation was approximately nine genes, as expected. The distribution of the number of falsely discovered genes has a long tail, but even so, only three of the 1,000 simulations generated more than 314 genes. This result indicates that it is highly unlikely that we would obtain 314 common age-regulated genes by sampling error.

We examined 401 gene sets defined by the Gene Ontology Consortium [16] to find those that exhibit age regulation in multiple tissues. We identified 84 gene sets (*p* < 0.001) that contained genes in which the overall age-related slopes trended either consistently up or down with age in multiple tissues (Table S6). At this threshold for significance, we would expect less than one gene set to appear age related by chance.

**Figure 1:**
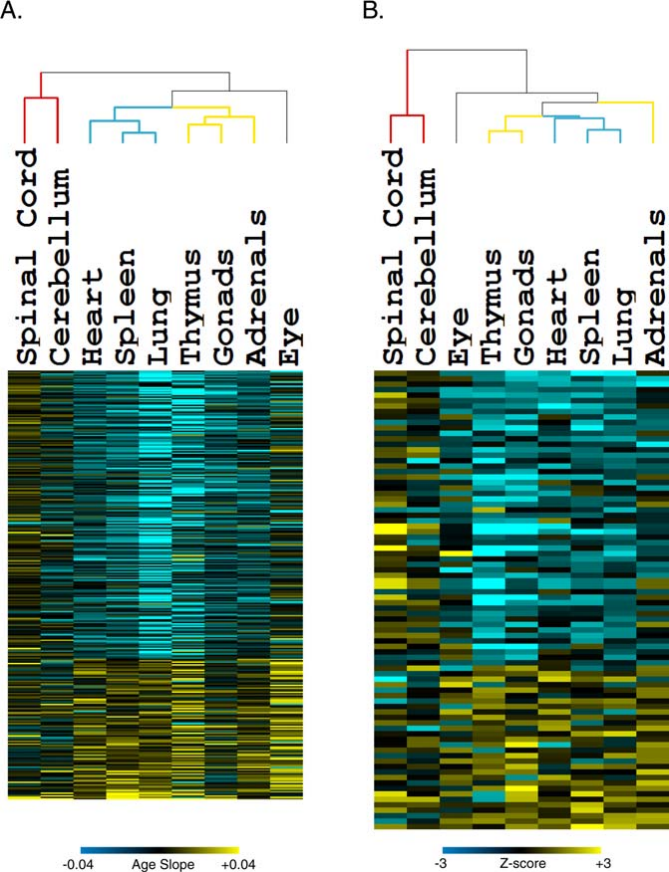
Genes and Gene Sets That Are Commonly Age Regulated in Multiple Tissues (A) Rows indicate individual genes, arranged from the gene that exhibits the greatest increase in expression with age across multiple tissues at top, to the gene that shows the greatest decrease in expression with age in multiple tissues at bottom. Each column corresponds to an individual mouse tissue. Scale corresponds to the slope of the change in log2 expression with age (*β_1j_*). Dendrogram colors indicate affiliation with either the neural (red), vascular (blue), or steroid-responsive (yellow) tissue groups. (B) Rows indicate individual gene sets, arranged from the gene set increasing expression most on average with age in multiple tissues at top, to the most decreasing with age. Each column corresponds to an individual mouse tissue. Scale is the van der Waerden *Z*-value as determined by gene set enrichment analysis. Dendrogram colors indicate affiliation with either the neural (red), vascular (blue), or steroid-responsive (yellow) tissue groups. A navigable version of this figure exists at http://cmgm.stanford.edu/~kimlab/aging_mouse.

To determine which tissues were aging similarly, we used the commonly age-regulated genes and gene sets to group tissues by unsupervised hierarchical clustering (Figure 1) [19]. Clustering tissues based on age regulation of genes as well as clustering based on gene sets showed three predominant patterns. The cerebellum and spinal cord clustered distinctly together as a neural group. The heart, lung, and spleen formed a second group of highly vascular tissues. The thymus and gonads were strongly grouped together in a steroid-responsive group. The adrenal glands clustered with this third group when clustering by genes, but not when clustering by gene sets. Finally, the eye remained an outlier; although it is considered neural tissue, it did not group with either the cerebellum or the spinal cord. In summary, these results identify three distinct modes of aging for different mouse tissues. Not all tissues age the same way, and different tissues may show different aging transcriptional profiles because they are subject to different forms of age-related stress.

**Figure 2:**
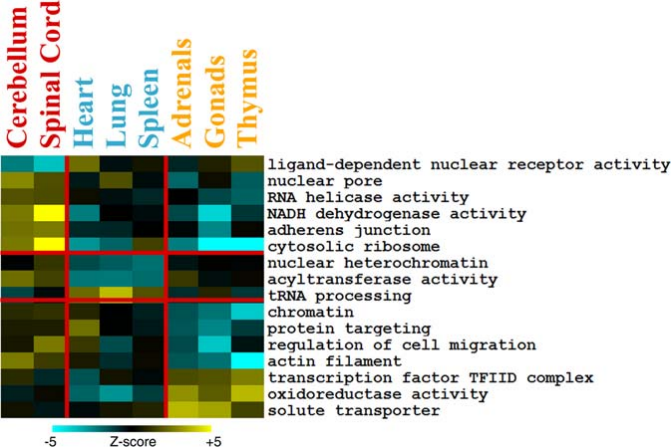
Differences in Age Regulation among Steroid-Responsive, Neural, and Vascular Tissues Columns refer to tissues, and rows correspond to gene sets. Vertical red bars divide tissues into neural, vascular, and steroid-responsive tissue groups from left to right. Tissue names are colored to indicate affiliation with either neural (red), vascular (blue), or steroid-responsive (yellow) tissue groups. Horizontal red bars divide gene sets into those chosen for being particularly age-regulated in steroid-responsive, neural, or vascular tissues (*p*-value < 0.001, all |*Z*| > 1.0, *p*-value > 0.5 in remaining two tissue groups) from top to bottom. Scale indicates van der Waerden *Z*-scores as determined by Gene Set Enrichment Analysis. A navigable version of this figure is available at http://cmgm.stanford.edu/~kimlab/aging_mouse.

To characterize the differences between the three different modes of tissue aging, we searched for gene sets that were strongly age regulated within one tissue group but not others. For each of the tissue groups, we first selected gene sets that showed strong age regulation in that tissue group (statistical significance at *p* < 0.001, and practical significance via a Van der Waerden *Z*-value with |*Z*| > 1), and then screened for those that showed much weaker age regulation in the other two tissues groups (*p* > 0.5) (Figure 2).

Genes in the cytosolic ribosome set stand out as the strongest age-related difference in expression between tissues. This gene set shows an increasing trend in expression in the neural group, but decreasing trends in the vascular and steroid-responsive groups. Increasing expression of ribosomal genes in neural tissues is curious, as rates of protein synthesis are known to decrease with age [20]. In addition to ribosomal gene expression, the neural aging group is characterized by age-regulated expression of genes encoding nuclear hormone receptors, NADH reductase enzymes, and adherens complex proteins. The nuclear hormone receptor gene set shows a decreasing trend in expression with age in neural tissues, which may be significant because age-related changes in expression of these transcription factors may also change expression in a battery of downstream genes. NADH reductase genes play an important role in the electron transport chain, and increased expression of these genes in neural tissues in old age may affect levels of oxidative damage. Neural tissues show increased expression trends of genes in the adherens junction gene set, which may affect communication between neurons in old age.

The steroid-responsive aging group shows decreased expression of genes in the protein targeting gene set. Genes in this gene set play an important role in protein secretion, suggesting a role for changes in hormone and growth factor expression in aging of steroid-responsive tissues.

Besides clustering tissues based on the expression profile of commonly age-regulated pathways, we also compared the degree of overlap in age regulation between each tissue with every other tissue. For each of the nine age-regulated mouse tissues, we selected genes that decrease expression with age, and genes that increase expression with age (*p* < 0.01). We calculated the extent of the overlap of age-regulated genes between all tissue pairings using Fisher's exact test for independence (Materials and Methods). We identified seven pairs of tissues that show a high degree of overlap (*p* < 0.001) (Table 2). These seven pairs form three groups of tissues that exactly match our tissue clustering results in Figure 1. Specifically, the spinal cord and cerebellum exhibit high levels of aging similarity. The heart, lung, and spleen show a great deal of overlap in age regulation with one another, as do the adrenal glands, thymus, and gonads. Interestingly, the eye shows a strong overlap of age-regulated genes with the heart, although the two tissues did not cluster together in Figure 1.

**Table 2:**
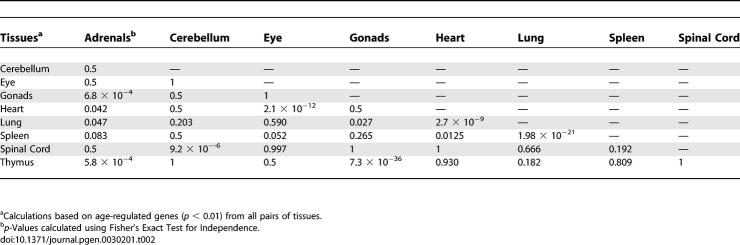
Similarities of Age Regulation for Nine Mouse Tissues

### Coordinated Aging of Different Tissues in Individual Mice

Individuals age at different rates, even when they have nearly identical genetic backgrounds and are grown under similar conditions. Different rates of aging can be seen by measuring the lifespan of individuals, the morphology and physiology of different tissues, or expression profiles of age-related genes. To what extent is the aging of one tissue coordinated with other tissues in the same individual? For instance, one might expect different organs in the cardiovascular system (heart and lung) to age in step with one another, but aging of the brain may not be tightly linked to aging of the heart. To test for coordination of aging among different tissues, we first developed a score for apparent age of each tissue in mice from the same age cohort based on the expression profile for that tissue. We then determined whether the apparent age of one tissue was correlated with the apparent age of another tissue in the same mouse.

To calculate an aging score for a specific tissue, we first use *Z*-normalized expression data from mice of every age to select the 50 genes that increase expression the most and the 50 genes that decrease expression the most with age in that tissue (Materials and Methods). The expression levels of these 100 genes are used to calculate the apparent age of that tissue in each individual mouse. For each tissue, we summed the relative expression levels for these 100 genes, generating a single value describing its apparent age for that mouse (Materials and Methods). We perform this operation for all nine tissues that show age-related changes in every mouse. Then, for every combination of age cohort and tissue, we assign each of the mouse samples a ranked fractional score on the basis of their age-regulated gene expression, with 1.00 representing the mouse with the oldest apparent age and 0.00 representing the youngest (Materials and Methods).

For each age cohort, we were interested in seeing whether there was coordinate aging among tissues. If aging is coordinated among tissues, then a mouse may contain many tissues with high apparent ages (represented by high fractional scores) and thus generate a very high overall aging score. Conversely, another mouse may contain many young tissues and generate a very small overall aging score. If aging is not coordinated among tissues, all mice should show a mix of old and young tissues, and thus have an overall aging score close to the average for all mice. We summed the fractional scores of all tissue for a given mouse within an age cohort, resulting in ten overall scores. Coordinate aging among tissues within an age cohort should generate a set of ten overall scores that shows a high variance, whereas independent aging should show a variance similar to those generated by random permutation. For each age cohort, we compared the variance of the ten overall scores to variances generated by permutation to calculate a *p*-value for coordinated aging (Materials and Methods).

**Figure 3:**
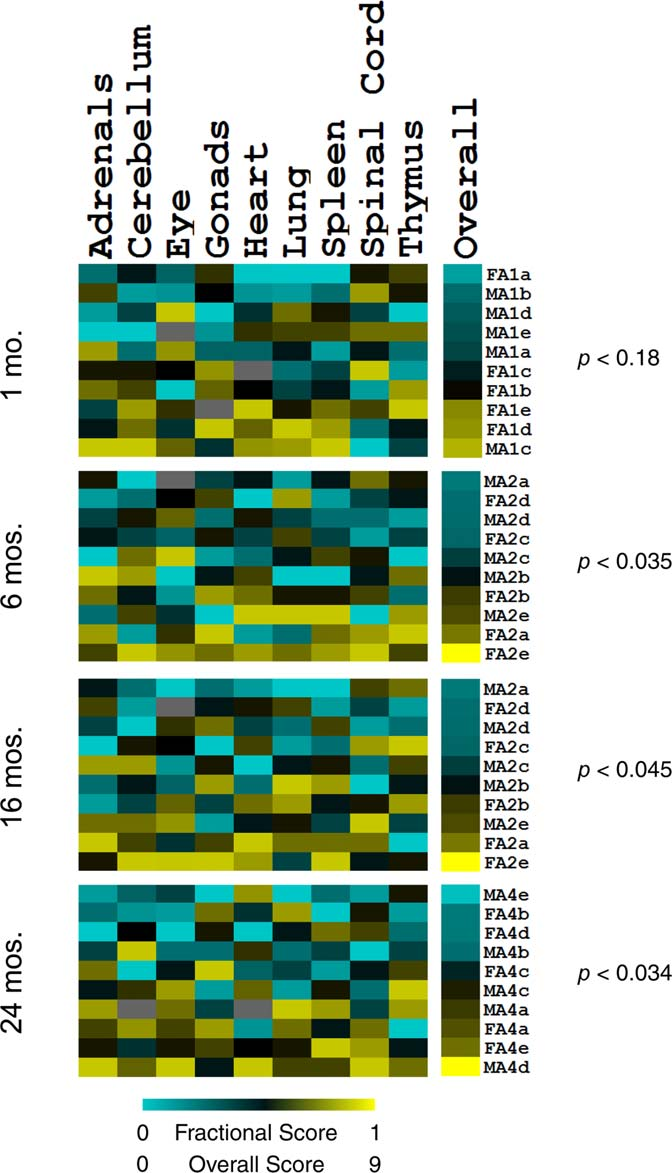
Coordinate Aging among Tissues in Individual Mice Columns refer to tissues. The rightmost column refers to the sum of fractional scores for each mouse. Rows refer to individual mice. Identification of individual mice indicates sex (M or F), age (A1 to A4), and biological repeat (a to e); e.g., FA1a is female mouse, age 1 mo, biological repeat a. Scale of tissue columns indicates the fractional score of that individual mouse with its tissue and age cohort; a low fractional score corresponds to a mouse that expresses age-regulated genes as a younger mouse, while a high fractional score expresses age-regulated genes as an older mouse. All fractional scores are summed for each mouse; the result is the overall score shown in the overall column. A low overall score corresponds to a mouse that expresses age-regulated genes as a younger mouse, while a high overall score denotes a mouse expressing age-regulated genes as an older mouse. Each *p*-value listed indicates the significance of tissue-coordinated aging for that age cohort.

At 6, 16, and 24 mo of age, we found that there is coordinate aging of different tissues (*p* < 0.05, Figure 3). Specifically, the variance of the sums of fractional scores was found to be significantly larger than the values generated from permuted data. The group of 24-mo-old mice showed the most coordination of aging among tissues.

### Common Age Regulation among Humans, Mice, Flies, and Worms

We wanted to find genes and gene sets that show similar age regulation in different species: M. musculus, H. sapiens, D. melanogaster, and *C. elegans*. To compare age regulation in similar microarray studies among these four species, we first identified orthologous genes (Table S7), and then we identified equivalent gene sets (GO gene groups comprised of between ten and 200 orthologous genes; Table S8). For each orthologous gene and gene set, we first used empirical meta-analysis to screen for age regulation in the nine mouse tissues shown in Figure 1 (*p* < 0.01). For each of these genes or gene sets, we next determined whether it also showed a similar pattern of age regulation in multiple human tissues (empirical meta-analysis; *p* < 0.01), using DNA microarray data on aging from human muscle [10], kidney [9], and brain [21]. Finally, we determined whether there were common patterns of aging in transcriptional profiles of aging in D. melanogaster [22] and C. elegans (Jiang et al., unpublished data) for orthologous genes (linear regression; *p* < 0.001) or gene sets (Gene Set Enrichment Analysis; *p* < 0.001).

**Figure 4:**
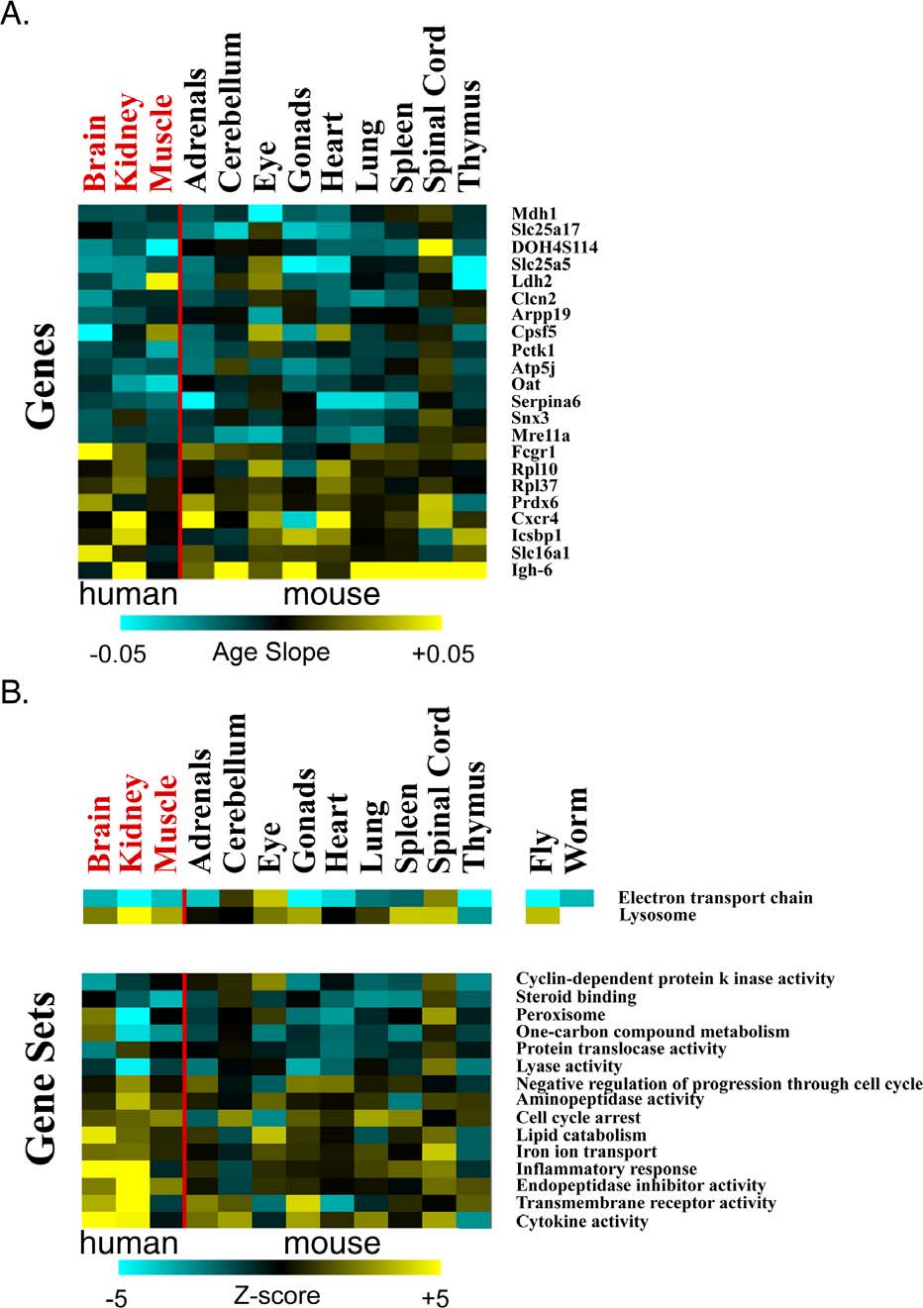
Common Age Regulation in Human, Mice, Flies, and Worms (A) Comparison of age-related expression changes for 2,578 orthologous genes in humans, mice, flies, and worms. Rows correspond to genes, arranged from the genes showing the greatest decrease in expression with age in both human and mouse at top to the genes showing the greatest increase in expression with age in both human and mouse at bottom. Columns correspond to tissues; tissue names are colored to denote their species of origin, either human (red) or mouse (black). Scale corresponds to the slope of the change in log2 expression with age (*β_1j_*). (B) Comparison of age-related expression changes for 280 gene sets in humans, mice, flies, and worms. First row is the mitochondrial electron transport chain gene set, which was found to be age regulated in flies and worms in addition to mammals. Second row is the lysosome gene set, which was found to be age regulated in flies in addition to mammals. Other rows correspond to gene groups that are commonly age related in humans and mice alone. Scale refers to a Van der Waerden *Z*-score. Columns correspond to tissues; tissue names are colored to denote their species of origin, either human (red) or mouse (black). A navigable version of this figure exists at http://cmgm.stanford.edu/~kimlab/aging_mouse.

This search yielded 22 genes and 17 gene sets that are commonly age regulated in mice and humans (Figure 4; Tables S9 and S10). Of these, only one gene set (the mitochondrial electron transport chain gene set) was commonly age regulated in all four species, showing an overall decreasing trend in expression in old age (*p* < 1 × 10^−13^ overall for four species). The mitochondrial electron transport chain is the primary source of free radicals in cells, which are highly reactive molecules that damage cellular components such as proteins and DNA. Overall reduction in expression of components of the electron transport chain may reduce levels of free radical damage in old age.

One other gene set (the lysosomal gene set) showed a common increasing trend in expression with age in humans, mice, and flies, but not worms. The lysosome is involved in degradation of extracellular proteins, and increased expression of lysosomal genes may reflect increased turnover of damaged cell surface proteins in old age.

Fifteen gene pathways show common age regulation in humans and mice but not flies or worms. The peroxisome gene set shows a decreasing trend in expression with age. The peroxisome is important for detoxification of foreign compounds and also produces hydrogen peroxide, a known precursor of free radicals. Decreasing expression of genes in the peroxisome pathway might lead to decreased tolerance toward toxins and decreased levels of oxidative damage in old age. The inflammatory response and cytokine activity gene sets show an increasing trend in expression with age, suggesting that inflammation becomes more active with age. Finally, genes involved in cell cycle arrest tend to increase expression with age, implying a tendency for cells to lose the ability to proliferate in the elderly.

**Figure 5:**
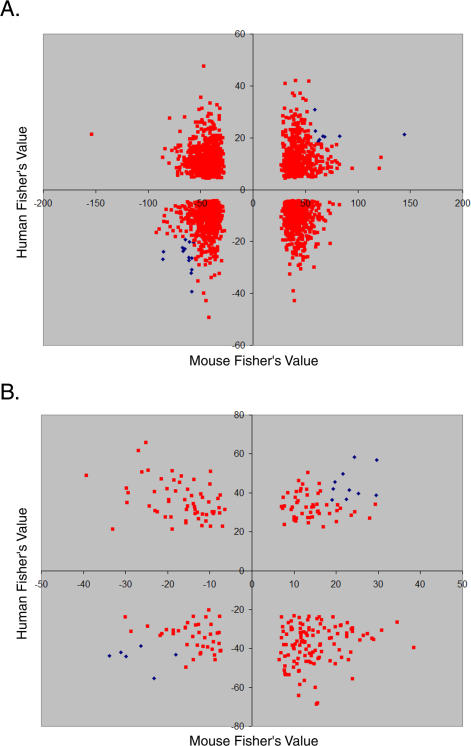
No Overall Correlation of Age-Regulated Expression Changes between Mouse and Human (A) Comparison of age-related expression changes for 2,578 orthologous genes in mouse and human. The *y*-axis indicates empirical meta-analysis *Z*-score for three human tissues (muscle, kidney, and brain). The *x*-axis shows the Fisher's meta-analysis value for nine age-regulated mouse tissues. Each gene had a *Z*-value for coordinated increases with age across tissues and another for coordinated decreases. We plot the more significant of the two here. Blue diamonds show genes that are age regulated to *p* < 0.01 in both human and mouse (*p* < 0.0001 overall). Red squares show all other genes. (B) Comparison of age-related expression changes for 280 gene sets in mouse and human. The *x*- and *y*-axes are as in A. Blue diamonds show gene sets that are age regulated to *p* < 0.01 in both human and mouse (*p* < 0.0001 overall). Red squares show all other gene sets.

Next, we determined whether there is an overall correlation between age-related expression in mice and in humans. That is, among the entire set of genes that increase expression with age in mice, is there also a tendency for these genes to increase expression with age in humans, and vice versa? As a measure of age regulation across nine mouse tissues and three human tissues, we used the *Z*-score from empirical meta-analysis. We then generated a scatter plot showing the Fisher's value for every orthologous gene and gene set in mice and humans (Figure 5A and 5B). We found that there was no correlation between age regulation in mice and humans and that the scatter plots were randomly distributed. Specifically, each gene that changed significantly in both mice and humans contributed to a 2 × 2 table recording whether it had a positive or negative *Z*-value in mice and similarly in humans. We computed Fisher's exact test for independence on that table and found no association at the level of genes (*p* < 0.15) and gene sets (*p* < 0.71). Genes or gene sets that increase with expression with age in mice may increase, decrease, or not change expression at all with age in humans. The same is true for genes or gene sets that decrease with expression with age. Finally, we repeated these analyses to compare aging in mice and humans for two specific tissues: kidney and muscle (Figure S1). As before, we found no overall correlation between age regulation in mice and humans. Thus, we have found no evidence for overall similarities in age-related expression between mice and humans.

## Discussion

We have provided an expression database on mouse aging to be used as a resource for the research community. This dataset is unprecedented in its magnitude and experimental uniformity; it contains a total of 5,519,976 expression values for 8,932 genes in 16 tissues at four times during aging with ten biological repeats at each time. The tissue samples were isolated from the same set of 40 mice, and were hybridized to similar gene arrays using identical protocols in the same lab to maximize uniformity. Out of practical consideration, the experiments were limited to the C57BL/6 strain, and future experiments will be needed to find age-related differences between C57BL/6 and other mouse strains. The full dataset can be downloaded (Table S1) and data for specific genes can be queried by researchers at http://cmgm.stanford.edu/~kimlab/aging_mouse and at http://www.grc.nia.nih.gov/branches/rrb/dna/agemap_data.htm. The DNA array experiments revealed a total of 906 age-regulated genes in nine different mouse tissues. These genes may be downstream markers for aging, such as stress response genes that are induced in old age due to accumulated oxidative damage. The expression levels of these genes inform us about the relative age of a tissue sample (i.e., high expression levels indicate high stress or old age). The identities of the age-related genes (i.e., stress response) provide important clues about mechanisms that drive transcriptional changes in old age (oxidative stress). This work identifies a large number of age-regulated genes that each provides an entry point for experimental study to determine its physiological role in aging. Further work may show that some genes are not only downstream markers, but that changes in their expression may either prolong life or hasten senescence. We note that in this work, we have analyzed these data using specific statistical techniques. Future analysis using increasingly advanced techniques may identify age-related behavior in gene expression that was not identified using current methods. We also focused on biological phenomena pertaining to changes in the mouse with age as a whole, as well as relationships and similarities between mouse tissues and between whole mouse and other species with age. In-depth work examining the effects of aging on specific, individual tissues or tissue groups has been accomplished for adrenal glands (S. Chigurupati, data not shown), central nervous system regions [23], thymus [24], and gonads (A. Sharov, data not shown).

In addition to revealing expression of one gene at a time during aging, the entire set of age-regulated genes can be used as a molecular phenotype of aging and may be used to assess the influence of antiaging interventions on the aging process. The expression levels from all of the age-regulated genes can be compiled into one score revealing the relative age of a tissue sample. One can view the age of a tissue at the molecular level using such a transcriptional profile of aging, similar to viewing the cellular age of tissues with a microscope or the age of individuals with a camera. We used the aging expression phenotype to discern whether aging has a great or small effect on specific tissues, to categorize different tissues into one of three distinct aging groups, and to compare molecular phenotypes of aging among different tissues and among different species.

Biomarkers of aging have been sought for some time for their potential utility in aging research [25]. In mice, numbers of T cells have been proposed to be measures of lifespan [26,27]. p16^Ink4A^ and other inhibitors of the cell cycle have been identified as a biomarker of aging in mice [28–30]. p16^Ink4A^ inhibits the cell cycle by repressing activity of cyclin-dependent kinases [31]. p16^Ink4A^ and other cell cycle inhibitors increase expression during aging, and may be markers of physiological age. The DNA array data presented here confirmed an increasing trend in expression with age of cell cycle inhibitors in mice, and further showed a similar increasing expression trend in humans suggesting that these may be biomarkers of human aging as well. In humans, gene expression profiles of aging in kidney and muscle can be used as aging biomarkers, as the expression profiles predict physiological age of tissues in different patients [9,10].

In this study, we identify gene expression profiles for nine different tissues in mice. Our results show a substantial heterogeneity in the amount of age regulation in diverse tissues. Nine tissues show some level of age-related expression changes, ranging from 17 to 346 age-regulated genes. Since the great majority of physiological changes in a tissue will also result in changes in expression (e.g., oxidative damage induces the oxidative damage stress response), we expect there to be a high correlation between those tissues that age the most and those that show the greatest changes in gene expression. Tissues with a large number of age-regulated genes (such as the thymus, eye, lung, and spinal cord) may be exceptionally prone to age-related changes, and thus may contribute a disproportionate amount to aging in the mouse overall. Age-related expression changes may be due either to changes in gene expression or to changes in cell heterogeneity within a tissue. For example, the thymus is known to involute in old age, as thymocytes are replaced by fat cells and the overall size of the thymus is dramatically reduced. The expression profile for aging in the thymus contains genes with high expression in fat cells that show increased abundance in the thymus in old age, and genes with high expression in thymocytes that show decreased abundance. Both changes in cell-intrinsic gene expression as well as changes in heterogeneous cell populations could functionally impact aging tissues, and we do not distinguish between the two in this work. Seven tissues showed little or no significant changes in expression with respect to age, despite having similar levels of technical variability as the rest of the tissues. The lack of transcriptional change with age indicates that these tissues may be exceptionally resilient to the aging process. However, aging may affect these tissues in ways that would not be detected by the gene arrays, such as changes in protein levels or amounts of oxidative damage.

Using the aging transcriptional profiles, we were able to cluster the tissues into three groups corresponding to three distinct modes of aging: neural, vascular, and steroid responsive. The formation of distinct aging clusters suggests that different tissues age via different pathways. The three distinct aging clusters might be affected to different extents in different mice, suggesting that individual mice may be composed of a mosaic of tissues with different physiological ages. However, these three age-related modes are defined by changes in gene expression and not by physiological measurements, and it will be important to determine whether these age-related transcriptional patterns accurately depict functional decline.

Age regulation of 14 gene sets distinguishes the different aging patterns of the three tissue groups. The strongest signature differentiating the three tissue groups involves genes that encode ribosomal subunits. Genes in this pathway decrease overall expression with age in the steroid-responsive and vascular tissues, but increase expression with age in neural tissues. This finding suggests a strong role for protein synthesis and ribosomal regulation in determining how different tissues age. Although the steroid-responsive and vascular tissues showed fairly similar patterns of age regulation, a chief difference between the two tissue groups was in the age-dependent regulation of oxidoreductase genes.

We have shown that many genes and several genetic pathways are age regulated in a similar fashion between human and mouse, despite the large disparity in lifespan between the two species. We have previously shown that the mitochondrial electron transport chain gene set shows an overall decreasing trend in expression with age in humans, mice, and flies [10]. Here, we have greatly expanded the analysis by including data from 15 more mouse tissues, and have shown that 22 genes and 17 gene sets change expression with age in both humans and mice. However, among all of these commonly age-regulated genes and gene sets, the mitochondrial electron transport chain gene set stands out because it is the only one that shows similar age regulation in flies and worms. One theory of aging is that accumulation of oxidative damage over a lifetime results in cellular senescence, and the electron transport chain is the primary source of oxidative damage via free radicals [32]. A decrease in expression level of genes in the electron transport chain pathway may reduce levels of oxidative damage in old age.

However, we found no global, overall correlation between age-related expression changes in mice with those from humans; we found similarity only in a few isolated, specific gene sets. Thus, an aging pathway that showed increased expression with age in mice is as likely to decrease with age in humans as it is to increase. Similarly, for pathways that decrease expression in mice. We performed this analysis by comparing the overall change in expression averaged from all nine mouse tissues and all three human tissues. We repeated the analysis by comparing age-related expression changes between mice and humans in single tissues (kidney and muscle). We found that none of these two tissues showed a significant correlation in age-related changes in expression between mice and humans (Figure S1).

The results from this paper indicate that age-related changes in a gene or pathway in mice do not reliably predict age-related changes in humans. Future experiments using a larger sample size, improved technology, or different analytical techniques may show an overall correlation between aging in mice and humans. Unless an overall correlation can be found, in order to understand human aging, it will be necessary to perform human experiments to find out which aspects of aging are shared between mice and humans. Once identified, focused experimental attention on these public age-related pathways in mice has the greatest potential to reveal mechanisms relevant to human aging.

## Materials and Methods

### Sample collection.

Male and female C57BL/6 mice were obtained from the National Institute on Aging (NIA) colony at 3 wk and 5, 15, and 23 mo of age. Mice were euthanized by cervical dislocation at 1, 6, 16, and 24 mo of age. Each mouse was dissected in a span of time ranging from 10–12 min. After cervical dislocation, mice were decapitated and an incision was made in the ventral side. Following the incision, the skin was degloved and harvested, exposing the abdominal tissues, which were harvested in order of pancreas, gonads, and liver. Following abdominal dissection, thoracic tissues were harvested in order of heart, lung, thymus, and kidney. Simultaneously with abdominal and thoracic dissection, the head was dissected and brain tissues (cerebellum, cortex, hippocampus, striatum, and spinal cord) collected. Finally, skeletal muscle and bone marrow were harvested last. All tissues were collected in RNAlater (Ambion) on ice and stored at −20 °C. The animal study protocol was reviewed and approved by the gerontology research center's animal care and use committee.

### RNA isolation.

All tissue samples were homogenized using a minibeadbeater-8 with 1.0mm zirconia/silica beads (Biospec Products). Total RNA was isolated from each sample homogenate using a RNeasy Mini Kit (Qiagen) in accordance with the manufacturer's protocol. RNA quality was assessed using an Agilent 2100 Bioanalyzer.

### Gene array hybridization.

All hybridizations were to NIA mouse 17K cDNA arrays. These arrays are composed of cDNA spots taken from both the NIA mouse 15K cDNA and NIA mouse 7.5K cDNA clone sets [33,34]. The 15K and 7.5K cDNA clone sets are constructed from E7.5 extraembryonic mouse tissue, preimplantation embryos, and stem cells using a PCR-based cDNA library construction method, and may therefore exclude some genes not expressed in placental and embryonic tissues. Each gene array membrane was prehybridized in 4 ml of hybridization buffer containing 3.2 ml of Microhyb (Invitrogen), 0.8 ml of 50% dextran sulfate, 100 μl of 10 mg/ml denatured human C_o_t DNA (Invitrogen), and 100 μl of 8 mg/ml denatured poly (dA) (Sigma-Aldrich) for 2 h at 55 °C. Radiolabeled cDNA probes were heat denatured and hybridized with gene array membranes using 4 ml of fresh hybridization buffer for 18 h at 55 °C. After three high-stringency washes in 2× SC and 0.1% SDS for 15 min at 55 °C, the membranes were exposed to a phosphor screen for 3 d and scanned using a Storm 860 PhosphoImager (Molecular Dynamics) with 50 μm resolution.

### Gene array normalization.

We calculated log_2_(expression level), and then used the *Z*-score method to normalize the gene array expression data as previously described [35]. All data will be available on the Gene Expression Omnibus upon publication, at http://cmgm.stanford.edu/~kimlab/aging_mouse, and at http://www.grc.nia.nih.gov/branches/rrb/dna/agemap_data.html. When different probes on the gene array corresponded to the same gene, we averaged the *Z*-scores from each of the probes together.

### Multiple regression analysis.

We used a multiple regression model to measure changes in expression with age for 8,932 genes in each of 16 individual tissues. This model assumed that differences in levels of gene expression may exist because of age or sex:

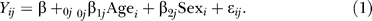
where *Y_ij_* is the expression level of the *j*th probe set for the *i*th sample, Age*_i_* is the age of the *i*th sample, Sex*_i_* corresponds to the sex of the *i*th sample (0 for male, or 1 for female), *ɛ_ij_* represents an error term, *β*_1*j*_ is the change of expression with age, *β*_2*j*_ is the change of expression with sex, and *β*_0*j*_ is the regression intercept. For each gene *j,* we used a least-squares approach to determine each coefficient; we were primarily interested in genes that show either a positive or negative value for *β*_1*j*_ *,* indicating either increasing or decreasing expression levels with age, respectively.

We wanted to determine whether the observed trends in expression of these genes were driven by developmental changes between 1-mo-old mice and older mice. We excluded the 1-mo-old cohort from the data, and then recalculated the slopes with respect to age for all of the genes that were originally found to be age related in Table 1. We found that all of the original age-related genes continued to show age-related trends in gene expression for 6–24 mo (unpublished data). This result indicates that the changes in expression occur throughout aging, and not only during development.

### Multiple comparisons control.

At *p* < 0.001, we would expect approximately nine genes on average to appear age regulated by chance alone, given a gene array containing 8,932 genes. If the gene tests were independent, then there would be less than a 10% chance of seeing 14 or more false rejections. However, expression levels of different genes are correlated for biological as well as technical reasons. This intrinsic biological correlation induces unperceived correlations in the test statistics for genes. To obtain an exceedance control that accounts for correlated genes we used permutations. We randomly permuted the ages of the 20 female mice and 20 male mice, maintaining sex. We then calculated the number of significant genes at *p* < 0.001 on each instance of permuted data. We repeated the permutation process 1,000 times and counted the fraction of those 1,000 repeats that generated more genes than we found in the original data. If that fraction was larger than 10% we did not consider the tissue to be age regulated.

### Gene set enrichment for regression.

We used a modified version of gene set enrichment analysis [14,15] to test whether gene sets either increase or decrease their overall gene expression levels with age [10]. Our modification replaces the weighted Kolmogorov-Smirnov test statistic by a Van der Waerden *Z*-score. The *Z*-score is intended to measure whether the bulk of the genes in the gene set are age regulated and is less affected by small numbers of extreme genes.

### Empirical meta-analysis of multiple mouse tissues.

For gene *j* and tissue *k* we computed a one-sided *p*-value 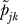 for age based on the *t-*test from a regression of gene expression on age and sex. The corresponding two-sided *p-*value is 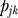 = 2min(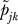 ,1 − 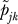 ). A standard method for combining *m* independent *p*-values is Fisher's method based on 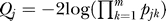 . When the *m p*-values are independent and all *m* null hypotheses hold, then 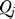 has the 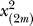 distribution.

Fisher's method is very powerful, but we needed to modify it. The statistic 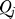 is equally sensitive to all 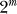 patterns in which gene *j* increases with age in one subset of tissues and decreases in another. To focus on genes that show consistent directionality, we introduce two one-sided meta analysis statistics, 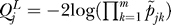 and 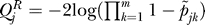 , which take extreme values for genes that are consistently decreasing or consistently increasing, respectively, over the *m* tissues. From these we obtained nominal one-sided *p*-values 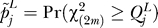 and 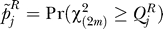 and the combined directionally sensitive *p*-value for gene *j* is 
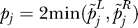
 .

The statistic 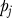 does not have the U(0,1) distribution even when gene *j* is unrelated to age in all *m* tissues. First, the factor 2 is a Bonferroni style correction for making two tests on that gene, and is somewhat conservative. Second, the *m* tissue-specific datasets on which 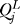 and 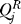 are based are statistically dependent because they have come from the same animals.

There were 346 genes with 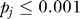 in the dataset. We discarded 32 of them for which the *p-*value was driven by essentially one tissue only, leaving 314 genes for further analysis.

The gene list is potentially too small because the factor of 2 may be too conservative and potentially too large because of dependencies among tissues. We validated the list empirically using the 8,932 observed genes, through a permutation analysis. In each resampling, the ages of the 20 male mice were randomly permuted, as were those of the 20 female mice. Male and female mouse ages were permuted independently. The newly generated ages were used for all tissues from the same animal. Among 1,000 such permutations, only three of them had more than the 314 significant genes found in the data.

Fisher's meta-analysis is perhaps the best-known method for combining multiple test statistics on a single hypothesis, but other approaches are available. Birnbaum [36] has shown that the most powerful combination method depends on the alternatives of interest. We constructed our test to be sensitive to alternatives where several alternatives hold with the same sign. Whitlock [37] mentions the combination method we have used in his introduction but discounts it in favor of some one-sided weighted *Z* alternatives to Fisher's test. Whitlock advocates a one-sided weighted *Z* test. Such a test would “punish” a gene heavily for having a slope in one tissue of a sign opposite to that which it has in several other tissues. We did not use *Z*-based tests because we are interested in finding such genes. We thank a referee for bringing Whitlock's paper to our attention.

### Analysis of tissue coordination.

For each age-regulated tissue, we first identified the 50 genes most increasing expression with age (as determined by slope with age) as well as the 50 genes with the greatest decrease in expression with age. For each tissue and mouse pair, we then calculated the expression:

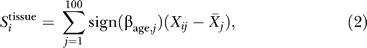
where S*_i_* denotes the score for the *i*th mouse, *β*_age,*j*_ shows the slope of expression with age for the *j*th gene, *X_ij_* is the Z-normalized expression level of the *i*th mouse in the *j*th gene, and 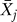 is the average Z-normalized expression level of the *j*th gene in the specific tissue. We then converted the raw scores for each tissue into fractional scores within each of the four age cohorts, denoted by:

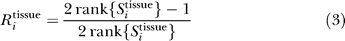



*R_i_* is the fractional score for the *i*th mouse. This method of generating fractional ranks minimizes the effects of missing mice for a particular tissue and age cohort by centering the fractional scores on 0.5 regardless of the number of mice-tissue pairs within an age cohort. We finally calculate the overall, or sum fractional, score by adding fractional scores across all tissues for each individual mouse as described by:

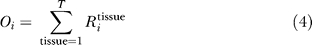



*O_i_* is the overall score for the *i*th mouse, and *T* is the total number of tissues; in this case, nine. In order to determine whether the overall scores for each of the four age cohorts were more widely distributed than we would expect by chance, we calculated the variance of the overall scores for all ten mice in each of the four cohorts. For each cohort, we then permuted the fractional scores for each mouse within each tissue 1,000 times, and recalculated overall scores using Equation 4 and determined the variance for each set of permuted fractional scores. The *p*-value for significance for each age cohort was equivalent to the number of times that permuted variances were greater than the actual variances.

We considered the possibility that the results could reflect a technical artifact in which the RNA from all of the tissues of an individual mouse was degraded, due to mishandling, disease, or some other factor. If so, then all of the transcripts from that mouse would show weak correlations with the other mice, not just the age-regulated genes. In order to address this possibility, we calculated the Pearson coefficients between individual mice within each tissue for all 8,932 genes. We did not observe any mice exhibiting an unusually low Pearson correlation with other mice (unpublished data), showing that the tissue coordination effect is seen for age-regulated genes.

## Supporting Information

Figure S1 No Overall Correlation of Age-Regulated Expression Changes between Mouse and Human in Kidney and Muscle (A) Comparison of aging coefficients for 106 genes found to be age regulated in human kidney in both human and mouse. The *x*-axis shows the change in expression with age for each of 106 genes in the aging human kidney data (scale is expression per year). Similarly, the *y*-axis shows the change in expression with age for the same genes in the aging mouse kidney data (expression per month). (B) Comparison of aging coefficients for 22 genes found to be age regulated in human muscle. The *x*-axis refers to aging coefficients in human muscle, while the *y*-axis refers to aging coefficients in mouse muscle.
(642 KB TIF)

Table S1 Aging Coefficients and *p*-Values for 8,932 Genes in 16 Mouse Tissues (7.9 MB XLS)

Table S2 Genes Age Regulated to *p* < 0.001 in Individual Mouse Tissues (361 KB XLS)

Table S3 401 Gene Sets Assayed for Age Regulation (50 KB XLS)

Table S4 Gene Sets Age Regulated to *p* < 0.001 in Individual Mouse Tissues (27 KB XLS)

Table S5 314 Genes Age Regulated in Multiple Mouse Tissues (197 KB XLS)

Table S6 84 Gene Sets Age Regulated in Multiple Mouse Tissues (40 KB XLS)

Table S7 2,578 Genes Assayed for Age Regulation in Human and Mouse (258 KB XLS)

Table S8 284 Gene Sets Assayed for Age Regulation in Human and Mouse (38 KB XLS)

Table S9 22 Genes Age Regulated in Common between Human and Mouse (18 KB XLS)

Table S10 17 Gene Sets Age Regulated in Common between Human and Mouse (19 KB XLS)

## Abbreviations

AGEMAP: Atlas of Gene Expression in Mouse Aging Project
