# Supplementary figures and images for: AGEMAP: A Gene Expression Database for Aging in Mice
Source: PLoS Genet. 2007 Nov 30;3(11):e201. doi: 10.1371/journal.pgen.0030201 (PMC2098796; doi:10.1371/journal.pgen.0030201)

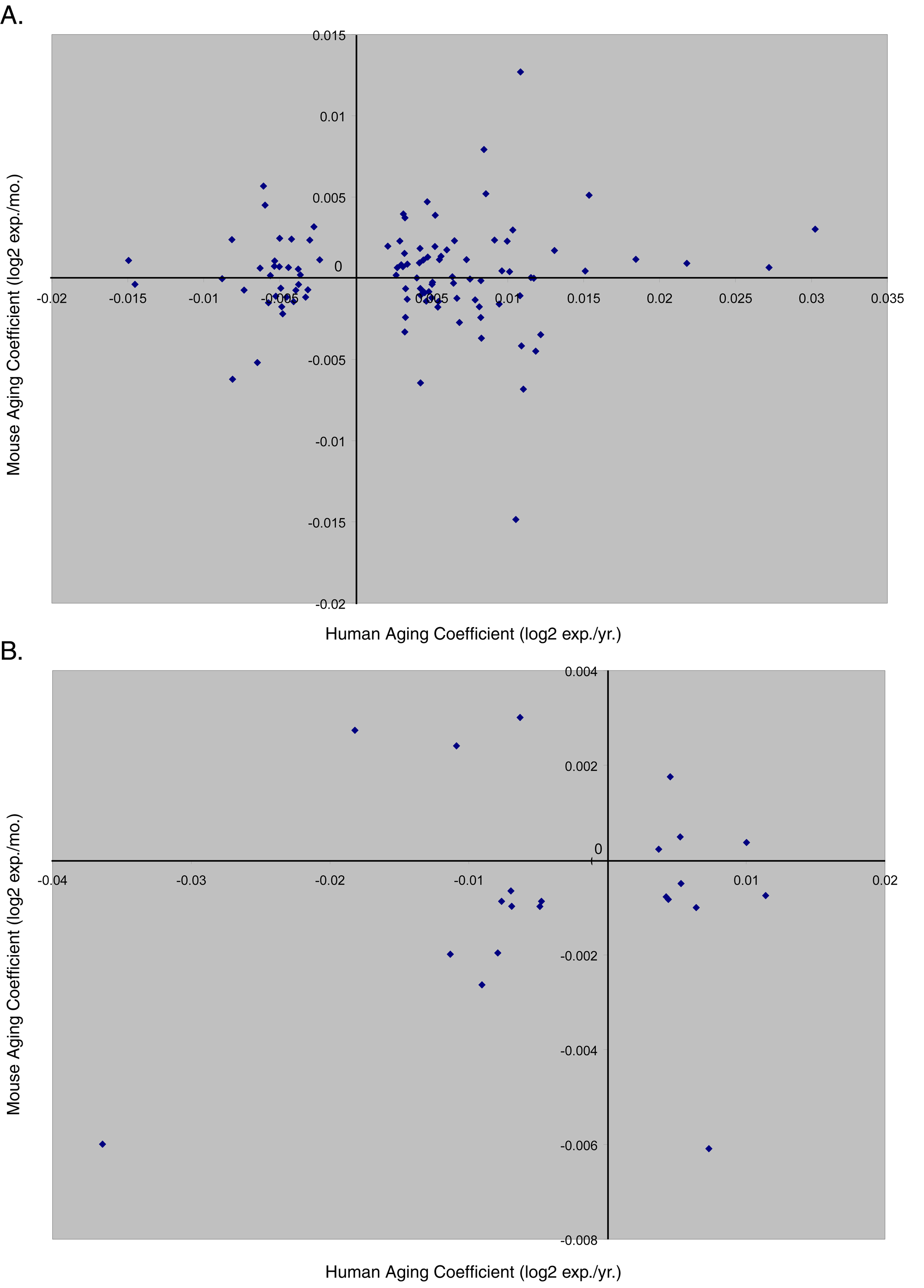

Supplement: Figure S1 — (A) Comparison of aging coefficients for 106 genes found to be age regulated in human kidney in both human and mouse. The x-axis shows the change in expression with age for each of 106 genes in the aging human kidney data (scale is expression per year). Similarly, the y-axis shows the change in expression with age for the same genes in the aging mouse kidney data (expression per month). (B) Comparison of aging coefficients for 22 genes found to be age regulated in human muscle. The x-axis refers to aging coefficients in human muscle, while the y-axis refers to aging coefficients in mouse muscle. (642 KB TIF) [file pgen.0030201.sg001.tif]
